# Supplementary material for: The Impact of Fixation on the Detection of Oligodendrocyte Precursor Cell Morphology and Vascular Associations
Source: Cells. 2021 May 24;10(6):1302. doi: 10.3390/cells10061302 (PMC8225113; doi:10.3390/cells10061302)
Supplement: Supplementary file 1 [file cells-10-01302-s001.zip › cells-1123223-supplementary.pdf]

supplementary Figure 1

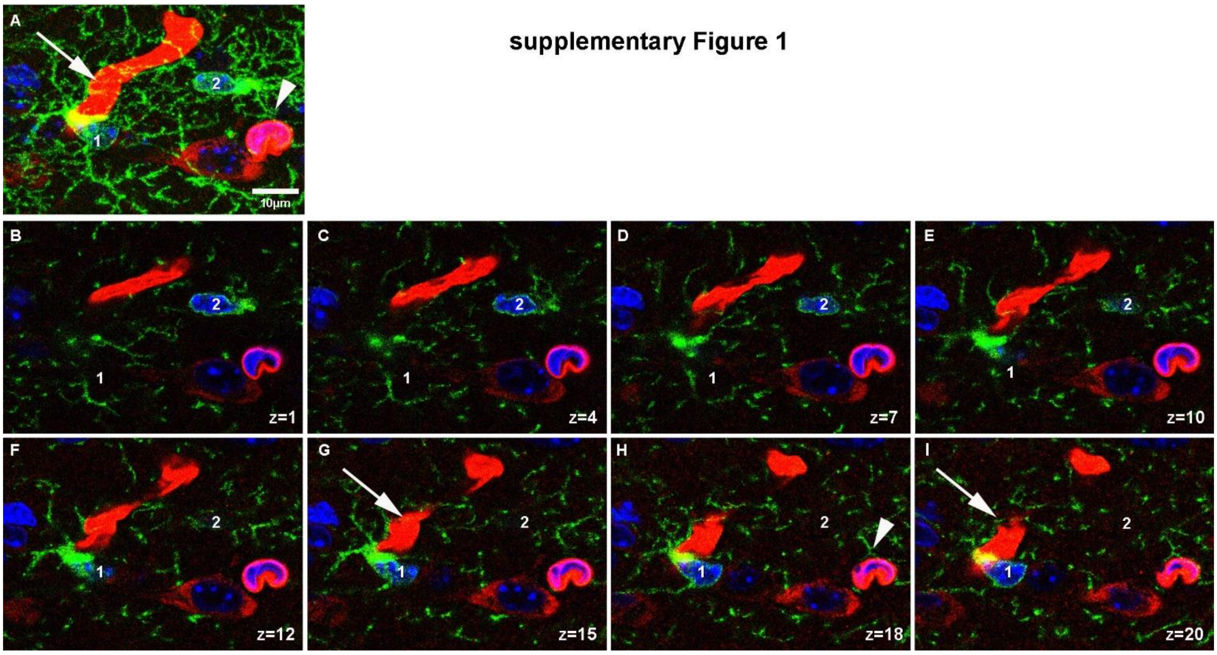

Supplementary Figure S1: OPC-blood vessel contacts

A region of interest showing two OPCs (indicated by 1 and 2 on the cell soma in white letters) each contacting another vessel segment in addition to the same vessel contacted by both cells. A maximum projection of 40 z-stacks is shown in A. Slices from the stack showing single protrusions contacting the surface of the vessel segments are shown in B-H. Arrow indicates contact made by OPC 1, arrowhead indicates contact made by OPC 2. Laminin-positive vessels are shown in red, PDGFRα-positive OPCs are shown in green, DAPI staining is shown in blue, position within the z-stack is indicated for each image

Scale bar represents 10µm.

supplementary Figure 2

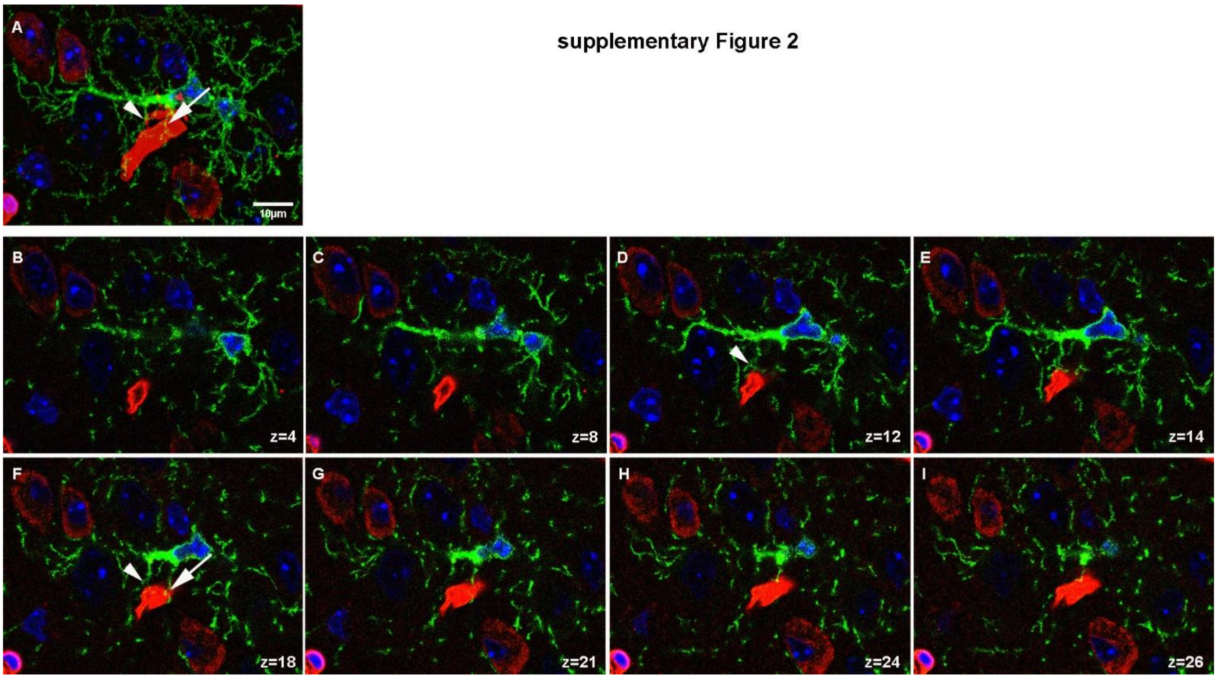

Supplementary Figure S2: OPC-blood vessel contacts

A region of interest showing an OPC doublet, of which the OPC on the left contacts the same vessel with 2 different protrusions (indicated by arrow and arrowhead). A maximum projection of 40 z-stacks is shown in A. Slices from the stack showing single protrusions contacting the surface of the vessel segments are shown in B-H. Arrowhead indicates contact made by protrusion 1, arrow indicates contact made by protrusion 2. Laminin-positive vessels are shown in red, PDGFRα-positive OPCs are shown in green, DAPI staining is shown in blue, position within the z-stack is indicated for each image.

Scale bar represents 10µm.

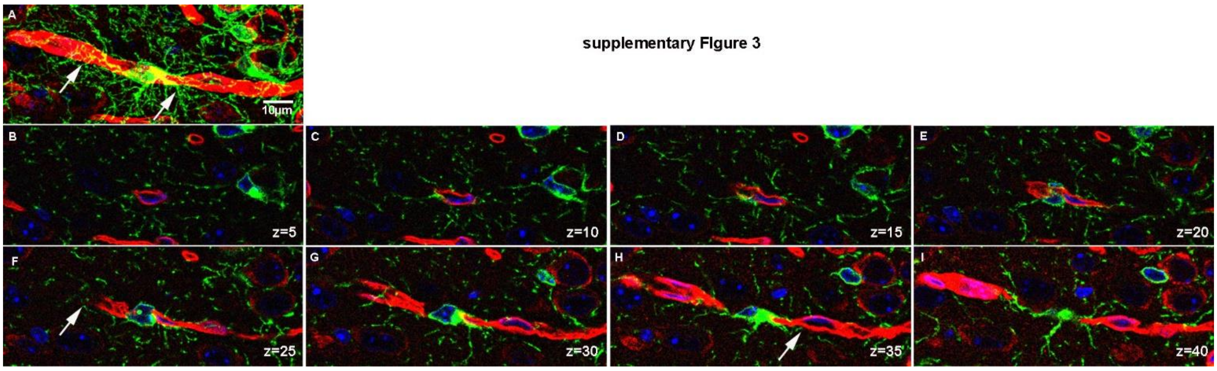

Supplementary Figure S3: OPC-blood vessel contacts

A region of interest showing an OPC with its soma localized directly adjacent to a vessel. A maximum projection of 40 z-stacks is shown in A. Slices from the stack showing single protrusions contacting the surface of the vessel segments are shown in B-H. Arrow indicates contact with the vessel made by protrusions of the OPC. Laminin-positive vessels are shown in red, PDGFRα-positive OPCs are shown in green, DAPI staining is shown in blue, position within the z-stack is indicated for each image.

Scale bar represents 10µm.

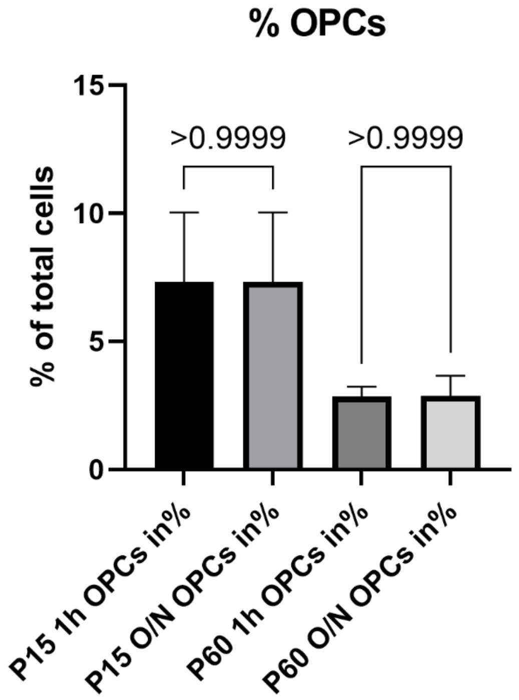

Supplementary Figure S4: Percentage of OPCs at P15 and P60

Percentage of OPCs among total DAPI+ cells detected by PDGFRα immunofluorescence after different fixation methods. There is no impact of the fixation protocol on the number of OPCs.
